# Sensitivity of methods for estimating breeding values using genetic markers to the number of QTL and distribution of QTL variance

**DOI:** 10.1186/1297-9686-42-9

**Published:** 2010-03-22

**Authors:** Albart Coster, John WM Bastiaansen, Mario PL Calus, Johan AM van Arendonk, Henk Bovenhuis

**Affiliations:** 1 Animal Breeding and Genomics Centre, Wageningen University, PO Box 338, 6700 AH, Wageningen, The Netherlands; 2 Animal Breeding and Genomics Centre, Animal Science Group, Lelystad, The Netherlands

## Abstract

The objective of this simulation study was to compare the effect of the number of QTL and distribution of QTL variance on the accuracy of breeding values estimated with genomewide markers (MEBV). Three distinct methods were used to calculate MEBV: a Bayesian Method (BM), Least Angle Regression (LARS) and Partial Least Square Regression (PLSR). The accuracy of MEBV calculated with BM and LARS decreased when the number of simulated QTL increased. The accuracy decreased more when QTL had different variance values than when all QTL had an equal variance. The accuracy of MEBV calculated with PLSR was affected neither by the number of QTL nor by the distribution of QTL variance. Additional simulations and analyses showed that these conclusions were not affected by the number of individuals in the training population, by the number of markers and by the heritability of the trait. Results of this study show that the effect of the number of QTL and distribution of QTL variance on the accuracy of MEBV depends on the method that is used to calculate MEBV.

## Background

In current breeding programs, estimation of breeding values is based on phenotypes of selection candidates and their relatives, often measured after animals reach to a certain age. This leads to a moderate to long generation interval, substantial costs and complex logistics for phenotypic recording [1]. Comparatively, breeding values estimated with genomewide distributed markers (MEBV) will increase annual genetic gain due to a reduced generation interval and improved accuracy, at lower costs [2,1].

Calculation of MEBV requires a population with information on genetic markers and phenotypes, called the *training *population. Phenotypic performance of the training population is used to estimate effects for the genetic markers which can be used to calculate MEBV of individuals with only marker information, called the *evaluation *population. Accuracy of MEBV depends on the heritability of the trait, the size of the training population, the method used to estimate marker effects and linkage disequilibrium (LD) between markers and quantitative trait loci (QTL) [2-6].

Linkage disequilibrium between markers and QTL is a function of the distance between markers and QTL and of the effective population size [7]. A large number of markers, distributed over the whole genome, is required to achieve high LD between markers and QTL when number and location of QTL on the genome are unknown. Simulation studies have shown that accuracy of MEBV increases when LD increases [2,8,9,4].

The accuracy of MEBV also depends on the variance of individual QTL since the ability to detect a QTL is related to its size. The size of a QTL, measured as the proportion of the genetic variance explained by that QTL, depends on its variance and on the genetic variance. Genetic variance, in turn, is a function of the number of QTL and of the variance of the individual QTL. Hayes and Goddard [10] have estimated parameters of a Gamma distribution describing the QTL effects found in published QTL detection experiments. This gamma distribution has been used in simulation studies to model the distribution of QTL effects [2,8,4,9,6]. Even though the distribution of QTL effects can vary considerably between different traits, the effect of the number of QTL on the accuracy of MEBV has been addressed only by Daetwyler [11] and the effect of distribution of QTL variance on the accuracy of MEBV has not been studied.

An important problem when estimating marker effects is the large number of markers relative to the number of phenotypes in the training data [2]. Meuwissen et al. [2] have solved this by using a Bayesian method (BM) that uses a sampling algorithm to obtain a posterior distribution of the marker effects. This Bayesian method is used in many simulation studies and in practical breeding programs, e.g. [12]. The Bayesian setup enables to incorporate a prior for the number of QTL and for the distribution of QTL effects [2]. Goddard [5] has found higher accuracies when a prior distribution for QTL effects reflecting the gamma (or exponential) distribution of QTL effects was used, compared to using a normal prior distribution for QTL effects. For many quantitative traits, however, the true distribution of the QTL effects is unknown.

Two other methods that might be suitable for estimating MEBV are Least Angle Regression (LARS) and Partial Least Square Regression (PLSR). LARS is a penalized regression method which identifies predictor variables that are highly correlated to the response variable and includes these in a regression model [13]. Park and Casella have shown similarities between LASSO, a variant of LARS, and Bayesian regression models [14]. They have shown that the posterior mode of a Bayesian model, similar to that proposed by Meuwissen et al. [2], and the regression coefficients estimated using LASSO are equal. Thus, LARS is a nonbayesian alternative to BM.

Regardless of the number of genetic markers, the rank of the matrix of marker data will be less or equal than the number of individuals in the training data. This implies the existence of correlations between marker genotypes. These correlations can be used to calculate MEBV by regressing the phenotypes on linear combinations of the markers. Partial Least Square Regression (PLSR) is a method that builds orthogonal linear combinations of the markers that have a maximum correlation with the phenotypes and regresses the phenotypes on these linear combinations, which are also called components [15]. Since components are orthogonal, regression coefficients of the components are independent. Datta et al. [16] have used PLSR in gene expression studies, Moser et al. [17] and Solberg et al. [6] have used PLSR to calculate MEBV.

Although BM and PLSR have been used independently to calculate MEBV, the accuracy of these methods when the number of QTL and the distribution of QTL variance varies is unknown. Therefore, the objective of this study is to investigate the effect of number of QTL and distribution of QTL variance on the accuracy of MEBV estimated with methods BM, LARS and PLSR.

## Methods

### Simulation of data

Each simulated genome consisted of four chromosomes of 1 Morgan each. Ten thousand loci were equally distributed over each chromosome, there were thus 40,000 loci distributed over the whole genome. In the base population, 4,000 of these loci, equally distributed over the genome, were made biallelic with allele frequency equal to 0.50. The remaining 36,000 loci were monomorphic in the base population. Two hundred gametes for the base population were simulated assuming linkage equilibrium and were randomly combined to create 100 individuals. Five thousand generations were simulated to generate LD between loci and to reach a mutation-drift equilibrium. Each individual in each generation contributed two gametes to the next generation with the objective of maintaining a population size of 100 individuals with Ne equal to 199 (the simulated population structure was thus different from a Wright-Fisher scenario). Each gamete transmitted to the offspring was simulated as an independent meiotic event. The number of recombinations for each chromosome was drawn from a Poisson(1) distribution, reflecting the size of the chromosomes in Morgan. The positions of the recombinations were sampled assuming no interference between recombinations.

Mutation rate for the 40,000 loci was set at 10^-5^. A mutation switched the allelic status; mutation of a 0 allele produced a 1 allele and mutation of a 1 allele produced a 0 allele.

Each individual in generation 5,000 contributed 10 gametes to generation 5,001, resulting in 50 fullsib families of 10 individuals each. Each individual in generation 5,001 contributed two offspring to generation 5,002, resulting in 250 fullsib families of 2 individuals each. Generation 5,001 was used as the training population and generation 5,002 was used as the evaluation population. Mutation rate was set to 0 in generations 5,001 and 5,002 to avoid the introduction of a large number of new alleles with a low Minor Allele Frequency (MAF). We simulated sixty replicates.

To simulate a range of QTL distributions, six scenarios were generated which were combinations of three levels for number of QTL and two distributions of QTL variance (Table 1). Depending on the scenario, up to fifty percent of the loci with a MAF greater than 0.10 were selected to become QTL in generation 5,001. QTL scenarios were numbered from 1 to 6, with increasing number of QTL accounting for 90% of the total genetic variance. Biallelic loci that were not selected as QTL in any scenario were used as biallelic markers. Within a replicate, this resulted in the same marker set across all QTL scenarios. Each QTL scenario was applied to all 60 replicates.

**Table 1:** Scenarios with different number of QTL and distribution of QTL variance.

Scenario	Number of QTL	Distribution of QTL variance
1	low	unequal
2	intermediate	unequal
3	high	unequal
4	low	equal
5	intermediate	equal
6	high	equal

Scenarios were numbered from 1 to 6, according to the number of QTL contributing 90% of the genetic variance.

The number of QTL contributing to the trait was changed by letting 5% (*low number of QTL*), 25% (*intermediate number of QTL*) or 50% (*high number of QTL*) of all loci with a MAF greater than 0.10 contribute to the trait. QTL for the scenarios with low and intermediate numbers of QTL were uniformly selected from the 50% of loci selected as QTL in the scenario with high number of QTL.

The variances of all QTL contributing to the trait were equal (*equal QTL variance*), or unequal (*unequal QTL variance*). The additive effects of QTL were calculated based on the specified QTL variance and the allele frequency of each QTL. For the scenarios of equal QTL variance, variance of each QTL was set to 1. For the scenarios of unequal QTL variance, variance of every tenth QTL was set to 81 and variances of the other 9 QTL were set to 1. In this way 10% of the QTL were responsible for 90% of the total additive genetic variance. The QTL effects were assigned to each QTL after the QTL were selected and therefore the same QTL were present in scenarios of equal and unequal QTL variance.

The true breeding value (TBV) of each individual was calculated as the sum of the allelic effects. Additive genetic variance, , was calculated as the variance of the TBV in generation 5,001. Deviates from a N(0, ) distribution were added to TBV and  was equal to  to simulate phenotypes with a heritability of 0.50.

In addition to the QTL scenarios, we studied the effect of heritability, pre-selection of markers based on MAF, and size of the training population on the accuracy of the MEBV calculated with the three methods. In the first alternative, heritability of the trait was reduced from 0.50 to 0.25. In the second alternative, markers with a MAF lower than 0.10 in the training population were excluded from the marker data. In the third alternative, the size of the training population was increased from 500 to 1,000 individuals by adding 10 fullsibs to each family while the size of the evaluation population was maintained at 500 individuals. Each alternative was applied to all six QTL scenarios and to the 60 replicates.

The simulations were performed with HaploSim [18], a package for R [19] which is available from the R repository CRAN http://cran.r-project.org/package=HaploSim. The simulations and computations were run on a system with a dual core Intel 2.33 Ghz processor and a Fedora Core 10 operating system.

### Analysis of population data

To validate and characterize the simulations, we determined the number of biallelic markers, heterozygosity of biallelic markers, linkage disequilibrium between adjacent markers and coefficient of determination of QTL. Heterozygosity of a population is the average number of heterozygous loci of an individual. Expected heterozygosity in a situation of mutation-drift equilibrium, expressed as a fraction of the total number of loci, is a function of mutation rate (u) and effective population size (Ne) [20]:(1)

In our simulations, where effective population size was 199 (Crow and Kimura, Equation 3.13.5 [20]) and mutation rate was 10^-5^, expected H is 7.90·10^-3^. For a genome consisting of 40,000 loci, the expected number of heterozygous loci in an individual is 316.

Linkage disequilibrium between adjacent markers was calculated as the squared correlation between adjacent markers and was expressed as r^2^.

The coefficient of determination of a QTL, expressed as R^2^, is the proportion of variance of that QTL explained by a set of markers. R^2^was calculated using the equation R^2 ^= c'K^-1^c, where c is a vector of correlation coefficients between the markers and the QTL, and K is the matrix of pairwise correlations of the markers. When the absolute correlation between a pair of markers exceeded 0.95, only one of these two markers was used to avoid singularity of matrix K. R^2 ^was calculated as the mean of R^2 ^between each QTL and the 50 markers in highest LD with that QTL and provided an estimate of the upper limit of the accuracy of MEBV that could be obtained based on this number of markers.

### Calculation of breeding values

We used three methods to estimate marker effects in the training population. The methods differed in how they estimated the additive effects of individual marker loci, but used an identical approach to calculate MEBV after these effects were estimated:(2)

where **MEBV **is the vector of breeding values estimated with the marker genotypes, **X **is an incidence matrix that relates genotypes to individuals, and **a **is the vector of additive effects for the markers, which is estimated by each method.

#### BM

The Bayesian Model (BM) used was proposed by Meuwissen and Goddard [2]. In this model, the additive effects of the markers are considered as independent random normal variables. The additive effect of markers which are considered to be associated to a QTL are sampled from a N(0, ) distribution. The additive effects of markers with are considered not to be associated to a QTL are sampled from a N(0, /100) distribution, which has a lower variance. The method requires a prior for the number of QTL and a prior for QTL variance . The prior for the number of QTL was set at 50 in all scenarios, regardless of the true number of QTL in that simulation scenario. The prior for QTL variance was set at 0.20, regardless of the simulation scenario.

BM uses Gibbs sampling to numerically integrate over the posterior distribution of the model. The sampler was run for 10,000 iterations and the first 1,000 iterations were discarded as burn-in. Regression coefficients of the markers were calculated as the means of their posterior distributions.

#### LARS

Least Angle Regression is a penalized regression method where predictor variables are included sequentially in the model [13]. Regression coefficients of all markers are zero at the start of the algorithm. LARS builds the model in sequential steps, in each step the marker that has the highest correlation with the residual is added to the model and the model proceeds in a direction of equal angle between all markers included in the model and the sequentially added marker [13]. After n steps, there are n markers in the model. We used the lars function in the lars package [21] of R and used cross validation on the training data to find the number of markers that minimized prediction error.

#### PLSR

Partial Least Square regression reduces the dimensions of the regression model by building orthogonal linear combinations of markers that have a maximal correlation with the response variable [15]. The trait is subsequently regressed on the linear combinations of markers, or components. Cross validation was used to find the number of components that minimized the prediction error.

To reduce the computation time required to fit the PLSR models, the algorithm to find the optimal number of components was modified as follows. In a first step, a model was fitted with ten components. Cross validation was used to find the optimal number of components. If the optimal number of components was below ten, this optimal number of components was used and the algorithm was stopped. If the optimal number of components was ten, a next iteration was performed with 20 components. If the optimal number of components, found by cross validation, was below 20, this number of components was used. Otherwise, the procedure was repeated with 30 components, and so on, until the number of components was equal to the number of observations or to the number of marker loci. The plsr function in the pls package [22] of R was used to fit and cross validate the models in each iteration. Cross validation was performed on the training data.

### Comparison of methods to calculate breeding values

The performance of each method was assessed based on the accuracy and the Mean Square Error of Prediction (MSEP) of MEBV. Accuracy of MEBV is the correlation between MEBV and TBV. Mean Square Error of Prediction is the average of the squared prediction errors of MEBV. Accuracy and MSEP were calculated based on individuals in the evaluation population.

Computation time of each method was recorded in all six QTL scenarios for ten replicates. The time recorded included the time required to fit the model on the training population, the time required for cross validation when using LARS and PLSR, and the time required to calculate MEBV for the evaluation population.

## Results

### Characteristics of simulated populations

Average heterozygosity was equal to 0.0110 in generation 1,000 and stabilized after 4,000 generation at 0.0076, corresponding to 304 heterozygous markers. This is slightly below the expected number based on Equation 1. The average number of biallelic markers in the data was 1,431 (Table 2). Eighty percent of these markers had a MAF below 0.10, reflecting an L-shaped distribution of MAF.

**Table 2:** Average (standard error) of number of polymorphic markers (nSNP), LD between adjacent markers (r^2^), number of QTL (nQTL), and average coefficient of determination of QTL (R^2^).

Situation	nSNP	**r**^**2**^	nQTL	**R**^**2**^
low nQTL	1431 (5.3)	0.048 (< 0.001)	35 (0.2)	0.806 (0.003)
low nQTL MAF > 0.10	374 (2.1)	0.145 (0.002)	35 (0.2)	0.715 (0.004)
int. nQTL	1431 (5.3)	0.048 (< 0.001)	172 (1.0)	0.811 (0.002)
int. nQTL MAF > 0.10	374 (2.1)	0.145 (0.002)	172 (1.0)	0.717 (0.002)
high nQTL	1431 (5.3)	0.048 (< 0.001)	343 (2.0)	0.811 (0.001)
high nQTL MAF > 0.10	374 (2.1)	0.145 (0.002)	343 (2.0)	0.717 (0.001)

The simulated number of QTL was low, intermediate (int.) or high and markers with a MAF lower than 0.10 were either or not included in the marker data. The table summarizes 60 replicated simulations.

Average LD between all adjacent markers, measured as r^2^, was 0.048 (Table 2). Expected LD, based on Equation 7 of Sved [7], is 0.31 (assuming an average distance between markers of 4/1431 Morgan). When markers with a MAF lower than 0.10 were excluded from the data, average LD between adjacent markers increased to 0.146 (Table 2). The expected LD based on Sved [7] is 0.11, however, does not account for mutations. To compare the LD obtained in our simulations with its expectation, we calculated the average LD between adjacent markers which were introduced in generation 0 and remained polymorphic in generation 5,000. On average, there were 174 of these markers and average LD between these markers was 0.036 which is close to the expected LD of 0.052 (assuming an equal distance between markers of 4/174 Morgan).

The average number of QTL was 35 in the scenarios with a low number of QTL and increased to 343 in the scenarios with a high number of QTL (Table 2). The average coefficient of determination of the QTL (R^2^) was 0.80 when all markers were used and 0.71 when markers with a MAF above 0.10 were used to calculate R^2 ^(Table 2).

Based on the average number of QTL (Table 2), the estimated number of QTL accounting for 90% of the total genetic variance ranged from 3, in scenario 1 (low number of QTL, unequal QTL variance), to 309, in scenario 6 (high number of QTL, equal QTL variance). The number of QTL accounting for 90% of the genetic variance in scenario 3 (high number of QTL, unequal QTL variance, approx. 31 QTL) was similar to that in scenario 4 (low number of QTL, equal QTL variance, approx. 35 QTL).

### Characteristics of MEBV

**Figure 1:**
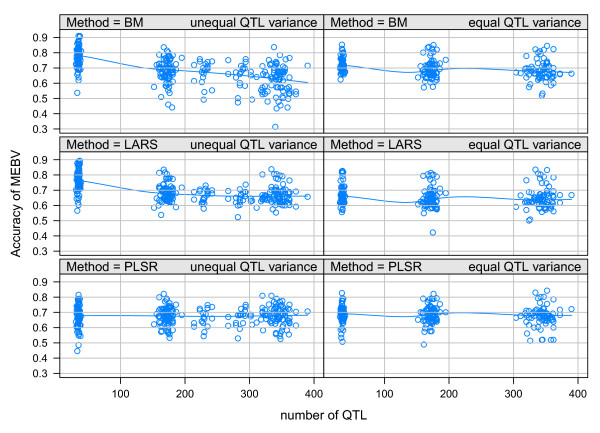
**Plot of the accuracies of MEBV calculated with BM, LARS and PLSR as affected by the simulated number of QTL**. The plots display the accuracies of 60 replicated simulations for number of QTL around 35, 172 and 343 plus the accuracies of 10 replicated simulation with number of QTL around 227 and 285 in the scenarios of unequal QTL variance. The variance of every tenth QTL was 81 times larger than variance of remaining QTL (unequal QTL variance) or equal for all QTL (equal QTL variance). The line is a LOESS smoother through accuracies on.

The average accuracy of MEBV calculated with BM and LARS decreased when the number of QTL increased and was stronger in the scenarios of unequal QTL distribution than in the scenarios of equal QTL distribution (Table 3 and Figure 1). The highest accuracies using BM and LARS were in scenario 1 (low number of QTL and unequal distribution of QTL variance) (Table 3). The highest accuracy using PLSR was in scenario 4, but with this method there was not a clear trend of accuracies between scenarios (see Table 3 and Figure 1). Overall, accuracies of BM were highest except in scenario 3 (Table 3).

**Table 3:** Average (standard error) accuracy of MEBV for individuals in the evaluation population.

Method	unequal QTL variance			equal QTL variance		
		
	low nQTL	int. nQTL	high nQTL	low nQTL	int. nQTL	high nQTL
	sc. 1	sc. 2	sc. 3	sc. 4	sc. 5	sc. 6
BM	0.77 (0.009)	0.67 (0.010)	0.60 (0.012)	0.71 (0.004)	0.67 (0.005)	0.67 (0.006)
LARS	0.75 (0.009)	0.67 (0.005)	0.65 (0.004)	0.65 (0.005)	0.63 (0.006)	0.63 (0.006)
PLSR	0.66 (0.009)	0.66 (0.007)	0.67 (0.007)	0.68 (0.006)	0.67 (0.006)	0.66 (0.007)

The MEBV were calculated with methods BM, LARS and PLSR. Simulated number of QTL was low (low nQTL), intermediate (int. nQTL) or high (high nQTL). The simulated variance of every tenth QTL was 81 times larger than variance of the remaining QTL (unequal QTL variance) or equal for all QTL (equal QTL variance). The averages and standard deviations were calculated using 60 replicated simulations.

Additional simulations were done with a number of QTL ranging between the intermediate and high number of QTL and using an unequal distribution of QTL variance to investigate the strong decrease of accuracies of BM from scenario 2 to scenario 3 (Table 3). Results of these additional simulations, confirm the decrease of accuracy of MEBV with BM between scenarios 2 and 3 (Figure 1).

The accuracy of MEBV decreased when heritability was reduced from 0.50 to 0.25 in the three methods (Table 4). In the scenarios with a low number of QTL (scenarios 1 and 4), BM was the most accurate (combining Tables 3 and 4). In the scenarios with an intermediate and high number of QTL, PLSR was the most accurate (combining Tables 3 and 4).

**Table 4:** Average (standard error) change of accuracy of MEBV for individuals in the evaluation population as affected by alternative simulation situations.

Method	unequal QTL variance			equal QTL variance		
		
	low QTL	int. QTL	high QTL	low QTL	int. QTL	high QTL
	sc. 1	sc. 2	sc. 3	sc. 4	sc. 5	sc. 6
**h^**2 **^= 0.25**						
BM	-0.14 (< 0.01)	-0.16 (0.01)	-0.08 (0.01)	-0.12 (< 0.01)	-0.16 (< 0.01)	-0.18 (< 0.01)
LARS	-0.14 (< 0.01)	-0.16 (< 0.01)	-0.15 (< 0.01)	-0.15 (< 0.01)	-0.14 (< 0.01)	-0.14 (< 0.01)
PLSR	-0.10 (< 0.01)	-0.11 (< 0.01)	-0.11 (< 0.01)	-0.11 (< 0.01)	-0.12 (< 0.01)	-0.11 (< 0.01)

**nTR = 1,000**						
BM	0.05 (< 0.01)	0.11 (0.01)	0.16 (0.01)	0.06 (< 0.01)	0.08 (< 0.01)	0.07 (< 0.01)
LARS	0.04 (< 0.01)	0.07 (< 0.01)	0.06 (< 0.01)	0.07 (< 0.01)	0.07 (< 0.01)	0.07 (< 0.01)
PLSR	0.07 (< 0.01)	0.06 (< 0.01)	0.06 (< 0.01)	0.06 (< 0.01)	0.06 (< 0.01)	0.07 (< 0.01)

**MAF **>**0.1**						
BM	-0.03 (< 0.01)	-0.01 (< 0.01)	0.02 (0.01)	-0.03 (< 0.01)	-0.03 (< 0.01)	-0.04 (< 0.01)
LARS	-0.02 (< 0.01)	0.00 (< 0.01)	-0.01 (< 0.01)	0.00 (< 0.01)	0.00 (< 0.01)	0.00 (< 0.01)
PLSR	-0.02 (< 0.01)	-0.01 (< 0.01)	-0.01 (< 0.01)	-0.03 (< 0.01)	-0.02 (< 0.01)	-0.01 (< 0.01)

Simulated heritability was reduced from 0.5 to 0.25 (h^2 ^= 0.25); the size of the training population was increased from 500 to 1,000 individuals (nTR = 1,000); only markers with a MAF above 0.10 were used to fit the models (MAF > 0.1). The simulated number of QTL was low (low nQTL), intermediate (int. nQTL) or high (high nQTL). The simulated variance of every tenth QTL was 81 times larger than variance of remaining QTL (unequal QTL variance) or equal for all QTL (equal QTL variance). Methods BM, LARS and PLSR were used to calculate the MEBV. The averages and standard deviations were calculated using 60 replicated simulations.

The accuracy of MEBV calculated with all methods increased when the size of the training population was increased from 500 to 1,000 individuals (Table 4) and BM was the most accurate method in all scenarios (combining Tables 3 and 4).

The accuracies of MEBV calculated with BM and PLSR decreased when markers with a MAF lower than 0.10 were excluded from the data, except for BM in scenario 3 (Table 4). Accuracies of MEBV calculated with LARS were not clearly affected by excluding markers with a MAF lower than 0.10. There was no clear effect of QTL scenario on the change of accuracies due to this exclusion (Table 4). The decrease of accuracies calculated with BM and PLSR when markers with a MAF lower than 0.10 were excluded was in line with the decrease of R^2 ^(Table 2).

Mean Square Error of Prediction of MEBV calculated with the three methods increased when the number of QTL increased (Table 5). The average MSEP of MEBV calculated with BM were low in all scenarios, except in scenario 3 where it was highest (Table 5).

**Table 5:** Average (standard error) of Mean Square Error of Prediction (MSEP) of MEBV for individuals in the evaluation population.

Method	unequal QTL variance			equal QTL variance		
		
	low nQTL	int. nQTL	high nQTL	low nQTL	int. nQTL	high nQTL
	sc. 1	sc. 2	sc. 3	sc. 4	sc. 5	sc. 6
BM	659 (26)	4049 (108)	10463 (343)	79 (2)	416 (6)	850 (12)
LARS	707 (24)	4019 (71)	8230 (124)	91 (2)	465 (6)	927 (12)
PLSR	993 (24)	4242 (73)	8405 (123)	93 (2)	458 (6)	922 (14)

Methods BM, LARS and PLSR were used to calculate the MEBV. The simulated number of QTL was low (low nQTL), intermediate (int. nQTL) or high (high nQTL). The simulated variance of every tenth QTL was 81 times larger than variance of remaining QTL (unequal QTL variance) or equal for all QTL (equal QTL variance). The averages and standard deviations were calculated using 60 replicated simulations.

The additive genetic variance increased when the number of QTL increased and was higher in the scenarios with unequal distribution of QTL variance (Table 6). This is due to the fact that the variance of 10% of the QTL was made 81 times larger than in the scenarios of equal QTL variance. The variance of MEBV calculated with the three methods was lower than the simulated additive genetic variance in all scenarios. The variance of MEBV calculated with PLSR was highest in all scenarios (Table 6). The variance of MEBV calculated with the three methods increased when number of QTL increased, except for the variance of MEBV calculated with BM in scenario 3 (Table 6). If MEBV were unbiased, then the variance of MEBV would be equal to r^2^, where r^2 ^is the squared accuracy of MEBV (Table 3). The variance of MEBV calculated with BM was lower than this expected variance in all scenarios (combining Tables 3 and 6). The variances of MEBV calculated with LARS and PLSR were higher than the expected variance in all scenarios and this difference was greatest for method PLSR (combining Tables 3 and 6).

**Table 6:** Average (standard error) of the simulated additive genetic variance () in the evaluation population, and variance of MEBV calculated for individuals in the evaluation population.

Method	unequal QTL variance			equal QTL variance		
		
	low QTL	int. QTL	high QTL	low QTL	int. QTL	high QTL
	sc. 1	sc. 2	sc. 3	sc. 4	sc. 5	sc. 6
	1623 (23)	7210 (88)	14193 (156)	158 (2)	767 (8)	1538 (18)
BM	890 (38)	2537 (168)	2032 (283)	81 (3)	327 (13)	575 (24)
LARS	914 (31)	3937 (164)	7017 (293)	75 (4)	344 (15)	715 (29)
PLSR	1249 (49)	5263 (198)	10747 (393)	129 (5)	618 (21)	1150 (46)

The methods BM, LARS and PLSR were used to calculate the MEBV. The simulated number of QTL was low (low nQTL), intermediate (int. nQTL) or high (high nQTL). The simulated variance of every tenth QTL was 81 times larger than variance of remaining QTL (unequal QTL variance) or equal for all QTL (equal QTL variance). The averages and standard deviations were calculated using 60 replicated simulations.

The average computation time required by the three methods increased when the size of the training population increased and when the number of markers included in the data increased (Table 7). In a normal situation, where the size of the training population was 500 individuals, all the markers were included in the data, and the heritability was equal to 0.50, PLSR required approximately 4 seconds to fit, cross validate and evaluate the models. LARS required approximately 211 seconds and BM required approximately 430 seconds (Table 7).

**Table 7:** Average (standard error) computation time required for fitting the MEBV models to the training population and calculating MEBV for the evaluation population, measured in seconds.

Method	Normal	h^**2 **^= 0.25	nTr = 1,000	MAF > 0.10
BM	423.25 (3.73)	429.57 (3.88)	820.75 (9.05)	109.49 (1.90)
LARS	211.75 (3.28)	210.92 (2.62)	1058.38 (9.34)	57.37 (1.80)
PLSR	4.05 (0.10)	4.10 (0.18)	6.47 (0.15)	0.81 (0.02)

Situation normal: heritability equal to 0.5, size of the training population equal to 500 individuals, and all markers included in the data. Situation h^2 ^= 0.25: heritability was decreased from 0.50 to 0.25. Situation nTr = 1,000: size of training population was increased from 500 to 1,000 individuals. Situation MAF > 0.10: markers with a MAF below 0.10 were excluded from the data. The table summarizes ten simulations for the scenario of intermediate number of QTL and equal QTL variance.

## Discussion and conclusions

The accuracies of MEBV calculated with the BM method in this study were compared to accuracies obtained by Calus et al. [4] and by Solberg et al. [6]. The approximate number of QTL was 75 in the simulations of Calus et al. [4], and 55 in the simulations of Solberg et al. [6]. Based on their descriptions, approximately seven QTL would account for 90% of the total genetic variance in both studies. Therefore, the simulations of Calus et al. [4] and Solberg et al. [6] are most comparable to scenario 1 (low number of QTL, unequal QTL variance), where an average of three QTL accounted for 90% of the total genetic variance.

The average accuracy of MEBV for individuals without performance data of their own by Calus et al. [4] was 0.75. The accuracy reported by Solberg et al. [6] in the scenario with a low number of markers was 0.69 with BM and 0.61 with PLSR. Accuracies in both studies, but especially in Solberg et al. [6], were lower than accuracies in scenario 1 of this study (Table 3). A lower LD between markers and QTL in the study of Solberg et al. [6] might be the reason for this lower accuracy.

The average LD between adjacent markers provides an indication for LD between markers and QTL because QTL are necessarily located somewhere between the markers. Average LD between adjacent markers can not be compared directly to expected LD based on Equation 7 of Sved [7] because mutations are expected to have a very strong impact on this LD. This strong impact is expected because a mutation will generally introduce a new marker between two markers which were previously considered adjacent. We calculated LD between adjacent markers that were polymorphic in generation 0 and still polymorphic in generation 5001. This LD can be compared to expected LD based on Sved [7] because newly mutated markers are not used and the effect of mutations on specific markers is negligible. Linkage disequilibrium, calculated in this way, was very similar to expected LD, providing evidence for the adequateness of our simulations.

Simulated QTL scenarios were numbered from 1 to 6, according to the number of QTL accounting for 90% of the genetic variance. The total number of biallelic QTL in the data is often used to describe simulations [3,4,9,6]; we think that the number of QTL accounting for a specific proportion of the genetic variance is a more appropriate description of the complexity of the genetic architecture underlying the trait. In this context, we expected similar results in scenarios 3 and 4 since the number of QTL accounting for 90% of the genetic variance were similar (34 in scenario 3 and 31 in scenario 4). Average accuracies of MEBV calculated with LARS and PLSR confirmed this expectation but accuracies with BM did not.

With method BM, higher accuracies were expected in QTL scenarios which more closely resembled the prior distributions for QTL number and distribution of QTL effects. The high accuracies with BM in scenario 1 were in line with this expectation but the stronger decrease of accuracies in scenarios 1 to 3 compared to the decrease of accuracies in scenarios 4 to 6 was not. The consistency of the decline in scenarios 1 to 3 was confirmed by additional simulations, with a number of QTL ranging between that in scenario 2 and in scenario 3. Accuracies of MEBV in these simulations confirmed this decrease (Figure 1).

To investigate whether accuracies of MEBV calculated with BM were affected by the prior distribution for QTL effects, we reanalyzed the data using a prior that more closely resembled the QTL scenarios that were simulated. In each scenario, the prior for number of QTL was set equal to the average number of QTL in this scenario (Table 2) and the prior for the variance of individual QTL was set equal to the average simulated genetic variance divided by the average number of QTL in this scenario (Tables 2 and 6). Comparison of these accuracies (Table 8) to the accuracies in Tables 3 and 4 shows that using a prior which is more correct does not improve average accuracy of MEBV. The accuracies of MEBV calculated with method BM in the different scenarios indicate that the highest accuracies are obtained with this method in situations were a small number of QTL accounts for a large proportion of the total genetic variance. The results in Table 8 indicate that the accuracies with BM did not depend on the correctness of the prior for QTL distribution and, furthermore, that a prior which was closer to the actual QTL distribution even led to lower accuracies in scenarios with a high number of QTL. These results contrast the results of Goddard [5], who found higher accuracies when a exponential prior for QTL effects was compared to a normal prior for QTL effects when the QTL effects were exponentially distributed. In this study, however, we compared accuracies obtained with different prior parameters, while using the same kind of distribution. Combining the results of Goddard [5] and of this comparison, it can be stated that using a correct kind of distribution as prior of QTL effects can be important for accuracy of BM but that the exact parametrization of this prior is not important.

**Table 8:** Average (standard error) accuracy of MEBV for individuals in the evaluation population.

Method	unequal QTL variance			equal QTL variance		
		
	low nQTL	int. nQTL	high nQTL	low nQTL	int. nQTL	high nQTL
Standard	0.80 (0.007)	0.67 (0.006)	0.57 (0.007)	0.69 (0.005)	0.62 (0.006)	0.57 (0.006)
h^2 ^= 0.25	0.68 (0.011)	0.52 (0.006)	0.56 (0.008)	0.57 (0.004)	0.51 (0.005)	0.53 (0.006)
MAF>0.10	0.77 (0.008)	0.69 (0.006)	0.64 (0.007)	0.67 (0.006)	0.66 (0.005)	0.61 (0.004)

The simulated number of QTL was low (low nQTL), intermediate (int. nQTL) or high (high nQTL). The simulated variance of every tenth QTL was 81 times larger than variance of remaining QTL (unequal QTL variance) or equal for all QTL (equal QTL variance). The rows of the table correspond to the standard situation (h^2 ^= 0.5, size of training population = 500 individuals, all markers included), the situation with h^2 ^= 0.25, and the situation where markers with MAF < 0.10 were excluded from the data. Method BM was used to calculate the. The prior number of QTL was 35 QTL in the scenarios with a low number of QTL, 172 QTL in the scenarios with an intermediate number of QTL, and 343 QTL in the scenarios with a high number of QTL. The prior for QTL variance was the ratio of the total genetic variance (Table 2) and the number of QTL. The averages and standard deviations were calculated using 60 replicated simulations

The number of QTL contributing to a trait is unknown in real situations. The scenarios of unequal QTL variance were motivated by the real situation where a few QTL contribute an important proportion of the total genetic variance. Examples of these situations include the DGAT1 gene and the SCD gene on bovine chromosomes 14 and 26 which contribute a large proportion of the genetic variation of milk fat content [23,24] and the IGF2 gene on porcine chromosome 2, which contributes a large proportion of the genetic variation of muscle mass in pigs [25]. Simulations and analyses that use a distribution similar to the one estimated by Hayes and Goddard [10] implicitly assume this situation. The scenarios with an equal QTL variance were motivated by the situations where many QTL contribute a small proportion of the total genetic variation of an individual trait, e.g. height in humans [26-28]. This study shows that accuracy and MSEP of distinct methods to calculate MEBV are affected by the distribution of QTL underlying a trait. Results of this study also show that the good performance of a method in one specific QTL scenario does not guarantee a good performance in other QTL scenarios.

Characteristics of the methods used to fit the MEBV models differed. Methods BM and LARS attempt to identify markers highly correlated with QTL and estimate effects for these markers. Results confirmed that the approach used by both BM and LARS, was advantageous when few QTL accounted for a large proportion of the total genetic variance. Method PLSR builds orthogonal, linear combinations of the predictor data (marker genotypes) that are highly correlated with the response and regresses the response on these components. The advantage of this method was that accuracies were almost not affected by the QTL scenario that was simulated; this was especially clear when comparing the decline of accuracies obtained with BM in scenarios 1 to 3 to the constant level of accuracies obtained with PLSR in scenarios 1 to 3 (Table 3). In this study, PLSR was advantageous over BM and LARS in situations where a large number of QTL contributed to the genetic variation of the trait of interest but methods BM and LARS performed better than PLSR in situations where few QTL contributed to the trait. An alternative method, not evaluated in this study, is GBLUP [2,29]. In this method, markers are used to estimate the relationship matrix of the individuals in the data and this relationship matrix is subsequently used to estimate breeding values with BLUP. Daetwyler [11] have reported that accuracy of GBLUP is not affected by the number of QTL in the data. In situations where few QTL contribute to the trait, accuracies obtained with BM are higher than accuracies obtained with GBLUP but at high number of QTL these accuracies are identical [11] suggesting that BM will always perform equally or better than GBLUP. When [5] derived accuracies for GBLUP and BM he showed that higher accuracy can be obtained with BM because this method better takes into account the variable contribution of individual QTL. Based on this, BM should be preferred over GBLUP. Since the number of QTL contributing to the trait is generally unknown, using the method PLSR can be a secure alternative for method BM. A pragmatic solution to overcome the problem of ignoring the number of QTL is cross validation [17]. For cross validation, a subset of individuals with highly reliable EBV can be used to evaluate the accuracy of MEBV obtained with BM, LARS and PLSR. The method which gives the highest accuracies can subsequently be used for the genetic evaluation of individuals with unknown breeding values.

Assignment of QTL by giving additive effects to biallelic loci was deferred to generation 5001. There were two reasons for not doing this earlier in the simulations. The first reason was to control the number of QTL that contributed to the trait. With QTL assigned in generation zero, the number of QTL will vary between replicates due to drift and mutations. The second reason was to reduce computing resources required for simulation. Simulating QTL is computationally more expensive than simulating loci because QTL require handling the additive effects in addition to the biallelic genotypes.

The six QTL scenarios were created after all generations were simulated, to ensure that QTL variance was the only difference between scenarios of equal and unequal QTL variance. The QTL scenarios were designed with the objective of identifying the effect of number of QTL and distribution of QTL variance on accuracy of MEBV with the distinct methods. A deterministic approach was used to assign the number of QTL contributing to the trait and to calculate the additive effect of each QTL contributing to the trait. This approach was very different from the random approach used to simulate QTL in other simulation studies (for example [2,30,4,9,6]) where QTL effects were drawn from a distribution similar to the gamma distribution for QTL effects estimated by Hayes and Goddard [10].

An important disadvantage of drawing QTL effects from any distribution is that randomness is introduced in the simulations that does not contribute to the research question because it is difficult to control the resulting distribution of QTL effects. The research question in our study concerned the effect of QTL distribution on the estimation of MEBV; hence distinct QTL scenarios covering a range of QTL distributions were simulated.

Strength of LD between a pair of loci is constrained by the difference between MAF of both loci [31]. In addition, variance of QTL with a low MAF is likely to be low, because the variance of QTL is a function of the allele frequency [32]. Excluding markers with a MAF below a specific threshold from the data, as done by Calus et al. [4], therefore seems reasonable. Results of this study, however, show that accuracy of MEBV was consistently lower when markers with a low MAF were excluded from the data (Table 4). These lower accuracies were supported by the lower R^2 ^when markers with a MAF below 0.10 were excluded (Table 2). Based on results of this study, using all markers to calculate MEBV is recommended.

This study reveals that method BM should be recommended in situations were few QTL are expected to account for a large proportion of the total genetic variance. When the number of QTL accounting for the genetic variance is larger or unknown, method PLSR is recommended.

## Competing interests

The authors declare that they have no competing interests.

## Authors' contributions

All authors were involved in the design of the study. AC and JB programmed the simulations and wrote the manuscript. All authors read and approved the manuscript.
